# Interim CDC Guidance for Polio Vaccination for Travel to and from Countries Affected by Wild Poliovirus

**Published:** 2014-07-11

**Authors:** Gregory S. Wallace, Jane F. Seward, Mark A. Pallansch

**Affiliations:** 1 Division of Viral Diseases, National Center for Immunization and Respiratory Diseases, CDC

In the prevaccine era, infection with wild poliovirus (WPV) was common worldwide, with seasonal peaks and epidemics in the summer and fall in temperate areas. The incidence of poliomyelitis in the United States declined rapidly after the licensure of inactivated polio vaccine (IPV) in 1955 and live oral polio vaccine (OPV) in the 1960s (1). The last cases of indigenously acquired WPV in the United States occurred in 1979, the last WPV case in a U.S. resident traveling abroad occurred in 1986, and the last WPV imported case was in 1993 (2,3). Since 2000, the United States has exclusively used IPV, resulting in prevention of 8–10 vaccine-associated paralytic poliomyelitis cases annually. In 2005, an unvaccinated U.S. adult traveling abroad acquired vaccine-associated paralytic poliomyelitis after contact with an infant recently vaccinated with OPV (4).

The Global Polio Eradication Initiative has made great progress in eradicating WPV, reducing the number of reported polio cases worldwide by >99% since the late 1980s. Only three countries remain in which WPV circulation has never been interrupted: Afghanistan, Nigeria, and Pakistan. However, polio could be brought into the United States from countries where WPV is circulating. During the last 6 months, 10 countries have had active transmission of WPV, and four of these countries have exported WPV to other countries. In the last 10 years, at least 40 polio-free countries have been affected through international travel (5).

In 2012, the completion of polio eradication was declared a programmatic emergency by the World Health Assembly (6). On May 5, 2014, the director-general of the World Health Organization (WHO) declared the international spread of polio to be a public health emergency of international concern under the authority of the International Health Regulations (7) and issued temporary vaccination recommendations for travelers from countries with active WPV transmission to prevent further spread of the disease (8). On June 2, 2014, CDC issued a health alert providing guidance to U.S. clinicians regarding new WHO polio vaccination requirements for travel by residents of and long-term visitors to countries with active poliovirus transmission (9). This report provides an update on CDC policy for polio vaccination of travelers for health protection. It also provides additional interim guidance for physicians whose U.S. resident patients will travel to or reside in affected countries for >4 weeks, to ensure those patients will have evidence of administration of polio vaccine (IPV or OPV) within 12 months of travel that might be required when they depart from countries with active poliovirus transmission. This interim guidance is to ensure compliance with WHO International Health Regulations temporary recommendations for countries designated as “polio-infected” to reduce the risk for exportation of WPV from those countries.

## Vaccine Recommendations and Requirements

Advisory Committee on Immunization Practices (ACIP) and CDC recommendations are evidence-based and provide public health recommendations to the general public on the basis of the best available epidemiological and scientific data to prevent poliovirus infection. This includes recommendations for travelers visiting countries with WPV circulation in the last 12 months or countries and provinces where they will be in situations with a high risk for exposure to persons with imported poliovirus infection.

Three countries are still endemic for polio (Afghanistan, Nigeria, and Pakistan). Countries where WPV has circulated during the previous 12 months include those endemic countries and those with polio outbreaks or environmental evidence of active WPV circulation during this time (Cameroon, Ethiopia, Equatorial Guinea, Iraq, Israel, Somalia, and Syria). Travelers working in health-care settings, refugee camps, or other humanitarian aid settings in these and neighboring countries might be at particular risk for exposure to WPV.

Recommendations for vaccination under the International Health Regulations differ from ACIP and CDC recommendations and include exit requirements for proof of polio vaccination when leaving the country at borders or through airports. If implemented by a country, these requirements could be mandatory and are intended to prevent exportation of WPV.

## Vaccine Recommendations for Travelers to Countries with WPV Circulation

Persons at greatest risk for acquiring polio are unvaccinated persons. In the United States, infants and children should be vaccinated against polio as part of a routine immunization series. Before traveling to areas with WPV circulation, all travelers should ensure that they have completed the recommended age-appropriate polio vaccine series and have received a booster dose, if necessary.*

### Infants and Children

In the United States, all infants and children should receive 4 doses of IPV at ages 2, 4, and 6–18 months and 4–6 years (10). The final dose should be administered at age ≥4 years, regardless of the number of previous doses**,** and should be given ≥6 months after the previous dose. A fourth dose in the routine IPV series is not necessary if the third dose was administered at age ≥4 years and ≥6 months after the previous dose (11). Infants and children traveling to areas where there has been WPV circulation in the last 12 months should be vaccinated according to the routine schedule. If the routine series cannot be administered within the recommended intervals before protection is needed, an accelerated schedule can be used as follows: 1) the first dose should be given to infants aged ≥6 weeks, 2) the second and third doses should be administered ≥4 weeks after the previous doses, and 3) the minimum interval between the third and fourth doses is 6 months.

If the age-appropriate series is not completed before departure, the remaining IPV doses to complete a full series should be administered when feasible, at the intervals recommended for the accelerated schedule. If doses are needed while residing in the affected country, the polio vaccine that is available (IPV or OPV) may be administered.

### Adults

Adults, who are traveling to areas where there has been WPV circulation in the last 12 months and who are unvaccinated, incompletely vaccinated, or whose vaccination status is unknown should receive a series of 3 doses: 2 doses of IPV administered at an interval of 4–8 weeks; a third dose should be administered 6–12 months after the second. If 3 doses of IPV cannot be administered within the recommended intervals before protection is needed, the following alternatives are recommended:

- If >8 weeks are available before protection is needed, 3 doses of IPV should be administered ≥4 weeks apart.
- If <8 weeks but >4 weeks are available before protection is needed, 2 doses of IPV should be administered ≥4 weeks apart.
- If <4 weeks are available before protection is needed, a single dose of IPV is recommended.

If <3 doses are administered, the remaining IPV doses to complete a 3-dose series should be administered when feasible, at appropriate intervals, if the person remains at increased risk for poliovirus exposure. If doses are needed while residing in the affected country, the polio vaccine that is available (IPV or OPV) may be administered.

Adults who have completed a routine series of polio vaccine are considered to have lifelong immunity to poliovirus, but data are lacking (12). As a precaution, persons aged ≥18 years who are traveling to areas where there has been WPV circulation in the last 12 months and who have received a routine series with either IPV or OPV in childhood should receive another dose of IPV before departure. For adults, available data do not indicate the need for more than a single lifetime booster dose with IPV.

## Interim Vaccination Guidance to Comply with WHO International Health Regulations Temporary Recommendations for Countries Designated as “Polio-infected”

U.S. clinicians should be aware of possible new vaccination requirements for patients planning travel for >4 weeks to the 10 countries identified by WHO as polio-infected (Figure) (13). Four countries (Cameroon, Equatorial Guinea, Pakistan, and Syria) are now designated as “exporting wild poliovirus.” Those countries should “ensure” recent (4–52 weeks before travel) polio boosters among departing residents and long-term travelers (of >4 weeks). An additional six countries (Afghanistan, Ethiopia, Iraq, Israel, Nigeria, and Somalia) are designated as “infected with wild poliovirus.” Those countries should “encourage” recent polio vaccination boosters among departing residents and long-term travelers. This list might change when the public health emergency of international concern is reassessed at the end of July, and, for some countries, these measures could extend beyond the 3 months validity of these temporary recommendations.†

Long-term (staying >4 weeks) residents of polio exporting or infected countries, including potential immigrants and refugees migrating to the United States, and travelers to those countries might be required to show proof of polio vaccination when departing the country. The polio vaccine must be received between 4 weeks and 12 months before the date of departure. As of June 12, 2014, Pakistan has implemented exit requirements for polio vaccination and the remaining exporting countries are expected to implement these requirements. The remaining countries with active WPV transmission might also implement exit requirements.

To ensure that U.S. travelers are properly prepared for any vaccination requirements they might face departing polio-exporting or polio-infected countries, CDC provides the following additional guidance:

- All polio vaccination administration should be documented on an International Certificate of Vaccination or Prophylaxis (often referred to as the WHO “yellow card”).§
- For children and adolescents who are up to date with IPV vaccination, including those who have completed the routine IPV series and who will be in a polio-exporting or polio-infected country for >4 weeks and their last dose of polio vaccine was administered >12 months before the date they will be departing that country, an additional dose of IPV should be given. Children who receive this additional dose as a fourth dose between ages 18 months and 4 years will still require an IPV booster dose at age ≥4 years.
- For adults with documentation of a polio vaccine series and an adult IPV booster dose who will be in a polio-exporting or polio-infected country for >4 weeks and their last dose of polio vaccine was administered >12 months before the date they will be departing that country, an additional dose of IPV should be given.
- If, before departure from the United States, the time residing in the polio-exporting or polio-infected country is anticipated to be >12 months, available polio vaccine (IPV or OPV) may be administered while in the affected country and 4 weeks to 12 months before departing that country.
- Clinicians performing overseas evaluations of immigrants and refugees migrating to the United States from polio-exporting or polio-infected countries should consult the 2014 Addendum to Technical Instructions for Panel Physicians for Vaccinations: Technical Instructions for Polio Vaccination for Applicants for U.S. Immigration for specific instructions.¶

## Vaccine Safety, Contraindications, and Precautions

Minor local reactions (pain and redness) can occur after IPV administration. No serious adverse reactions to IPV have been documented; however, experience with administration of multiple additional doses is limited. IPV should not be administered to persons who have experienced a severe allergic reaction (such as anaphylaxis) after a previous dose of IPV or after receiving streptomycin, polymyxin B, or neomycin, which IPV contains in trace amounts. Hypersensitivity reactions can occur after IPV administration among persons sensitive to these three antibiotics. If a pregnant woman is unvaccinated or incompletely vaccinated and requires immediate protection against polio because of planned travel to a country or area where polio cases are occurring, IPV can be administered as recommended for adults. Breastfeeding is not a contraindication to administration of polio vaccine to an infant or mother (10,12).**

## Figures and Tables

**FIGURE:**
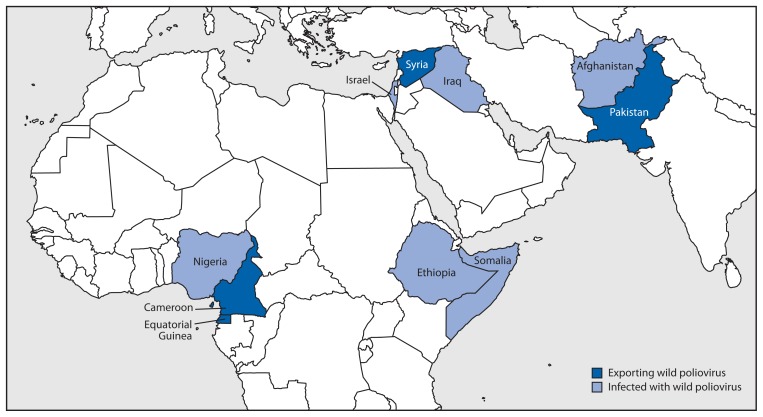
Countries identified by the World Health Organization as exporting wild poliovirus and those currently wild poliovirus–infected — worldwide, 2014* * As of June 30, 2014.

## Footnotes

* Full information on ACIP recommendations for poliomyelitis vaccination is available at http://www.cdc.gov/vaccines/hcp/acip-recs/vacc-specific/polio.html. Additional information is available at http://wwwnc.cdc.gov/travel/yellowbook/2014/chapter-3-infectious-diseases-related-to-travel/poliomyelitis. Vaccine recommendation information for specific countries is available at http://wwwnc.cdc.gov/travel/destinations/list.

† Updates will be available at http://wwwnc.cdc.gov/travel/page/clinician-updates.

§ Additional information on the International Certificate of Vaccination or Prophylaxis is available at http://wwwnc.cdc.gov/travel/yellowbook/2014/chapter-3-infectious-diseases-related-to-travel/yellow-fever#2849.

¶ Available at http://www.cdc.gov/immigrantrefugeehealth/exams/ti/panel/technical-instructions-panel-physicians.html.

** Additional information on precautions, contraindications, and immunization of persons with immunosuppression and their household contacts is available at http://www.cdc.gov/vaccines/vpd-vac/polio.
